# Temperature-Dependent Photoluminescence of ZnO Thin Films Grown on Off-Axis SiC Substrates by APMOCVD

**DOI:** 10.3390/ma14041035

**Published:** 2021-02-22

**Authors:** Ivan Shtepliuk, Volodymyr Khranovskyy, Arsenii Ievtushenko, Rositsa Yakimova

**Affiliations:** 1 Department of Physics, Chemistry and Biology, Linköping University, SE-581 83 Linköping, Sweden; volodymyr.khranovskyy@liu.se (V.K.); rositsa.yakimova@liu.se (R.Y.); 2 Department of Physics and Technology of Photoelectronic and Magnetoactive Materials, I. Frantsevich Institute for Problems of Material Science, NASU, 3 Krzhizhanovskogo str., 03142 Kyiv, Ukraine; earsen@ukr.net

**Keywords:** ZnO, SiC, off-cut angle, luminescence, APMOCVD

## Abstract

The growth of high-quality ZnO layers with optical properties congruent to those of bulk ZnO is still a great challenge. Here, for the first time, we systematically study the morphology and optical properties of ZnO layers grown on SiC substrates with off-cut angles ranging from 0° to 8° by using the atmospheric pressure meta–organic chemical vapor deposition (APMOCVD) technique. Morphology analysis revealed that the formation of the ZnO films on vicinal surfaces with small off-axis angles (1.4°–3.5°) follows the mixed growth mode: from one side, ZnO nucleation still occurs on wide (0001) terraces, but from another side, step-flow growth becomes more apparent with the off-cut angle increasing. We show for the first time that the off-cut angle of 8° provides conditions for step-flow growth of ZnO, resulting in highly improved growth morphology, respectively structural quality. Temperature-dependent photoluminescence (PL) measurements showed a strong dependence of the excitonic emission on the off-cut angle. The dependences of peak parameters for bound exciton and free exciton emissions on temperature were analyzed. The present results provide a correlation between the structural and optical properties of ZnO on vicinal surfaces and can be utilized for controllable ZnO heteroepitaxy on SiC toward device-quality ZnO epitaxial layers with potential applications in nano-optoelectronics.

## 1. Introduction

There is a long-standing interest in implementing zinc oxide (ZnO) into realistic optoelectronic applications [1,2,3,4,5,6,7,8]. Numerous existing theoretical and experimental studies were mainly triggered by its unique optical properties, especially large exciton binding energy (60 meV) at room temperature (RT), which is even higher than that of GaN. This makes ZnO an excellent candidate for the next-generation ZnO-based quantum well light-emitting devices [9,10,11,12,13]. Naturally, the ultimate development of ZnO-based technologies requires the production of device-quality ZnO layers with tunable band gap energy, controllable doping level, low defect density, minimized strain, and high optical performance (good transparency and strong light emission). One of the possible strategies to satisfy such strict requirements is the homoepitaxy of ZnO on native bulk ZnO substrates [14,15,16]. Nevertheless, up to now, no cheap ZnO bulk substrates combining high crystal quality, low defect concentrations, and large size are commercially available. For this reason, high-quality ZnO crystalline layers have been typically fabricated using the heteroepitaxy approach via growth on foreign substrates with suitable structural and thermal properties [17,18,19,20]. Among such substrates, silicon carbide (SiC) is an eligible candidate, since its growth technology provides high crystal quality over a large-scale substrate size [21]. SiC possesses also high thermal conductivity [22], which is a predominant criterion for heat distribution over the substrate or wafer during the growth processes. Considering that the ZnO has a larger thermal expansion coefficient (5.36 × 10^−5^ K^−1^ at RT [23]) than that of SiC (3 × 10^−6^ K^−1^ at RT for 6H-SiC [24] and 3.1 × 10^−6^ K^−1^ at RT for 4H-SiC [25]), there is a possibility to grow unstrained high-quality ZnO epilayers with a low density of linear and planar defects (dislocations, stacking faults, cracks) and advanced luminescence properties [26]. However, a large in-plane lattice mismatch (≈5%) of ZnO and SiC makes direct epitaxial growth rather challenging. Indeed, lattice mismatch as small as ≈2% can be already a severe issue for high-quality heteroepitaxy, while in the case of larger lattice mismatch (≥2%), the overgrown material turns into a polycrystalline film eventually. Hence, a possible solution to diminish the problem of dislocation generation due to lattice mismatch is using a vicinal surface of misoriented SiC substrates [27]. Earlier, it was demonstrated that this approach enables epilayer growth via the step flow mechanism [28,29], which limits the generation of threading dislocations [30]. This is due to a higher step surface density of the off-cut angle substrates, which improves the nucleation of thin films. In this regard, the improved structural properties of layers grown on vicinal surfaces will be translated into the enhanced optical performance. 

For the sake of completeness, it should be noted that numerous deposition techniques have been exploited for the growth of heteroepitaxial ZnO films, including radio frequency (rf) or direct current (dc) magnetron sputtering [31,32], molecular beam epitaxy [33], pulsed laser deposition [34], atomic layer deposition [35], electrodeposition [36], thermal evaporation [37], sonochemical synthesis [38], microwave-assisted wet chemical growth [39], hydrothermal synthesis [40], sol–gel [41], and metal–organic chemical vapor deposition (MOCVD) [42,43]. Among the listed techniques, MOCVD has been broadly recognized as one of the most reliable approaches to grow device-quality optoelectronic materials, which is driven by its most obvious advantages in terms of scalable production, easier compositional control, doping efficiency, higher growth rate, and better step coverage. In most cases, the MOCVD process occurs under low-pressure conditions, which enables preventing the undesired gas phase reactions and defects generation. However, as was experimentally shown for gallium nitride films [44,45], compared to the low-temperature process, the atmospheric pressure MOCVD can be a more profitable approach to synthesize films with larger grain size and higher carrier mobility. This is explained by the fact that the number of nucleation sites on the substrate surface in the case of atmospheric pressure growth is much lower than that for low-pressure growth. The reduced nucleation density favors the lateral growth and the formation of larger grains, which are important prerequisites for the growth of high-quality epitaxial ZnO layers [46]. It seems that some trade-off between pressure, defect density, and nucleation density must be reached to make MOCVD-grown ZnO films more applicable. In this regard, the utilization of vicinal surfaces of SiC as substrates in APMOCVD growth may provide significant additional leverage to control ZnO nucleation and hence ZnO optical properties due to the possibility of tuning the relationship between step density and terrace width. Considering potential applications, it is particularly important to correlate the structure quality of as-grown ZnO layers on vicinal SiC substrates with their optical properties, especially ultraviolet (UV) excitonic emission. From the practical point of view, understanding excitonic behavior is imperative to govern luminescent properties of the ZnO component in realistic devices. In this respect, the nature of UV near-band edge (NBE) emission from ZnO films grown on vicinal surfaces of SiC substrates (with different off-cut angles) has not been systematically reported in the literature so far. Thus, the main aim of this work is to reveal the effect of the vicinal surface of the SiC substrate on the emission mechanism of ZnO films via modified growth mode. Bearing in mind that structural imperfections and defects are known to deteriorate the excitonic emission in ZnO, one of the most relevant challenges here is to define the optimal off-axis angle that has a beneficial impact on ZnO crystal quality. In this context, temperature-dependent photoluminescence spectroscopy, which is highly sensitive to material quality and defect density, is a powerful tool to provide a deep comprehension of the excitonic emission in ZnO films grown on the vicinal surfaces of SiC. 

In this study, we outline and compare the morphology and luminescent properties of ZnO films grown on vicinal SiC (0001) surfaces with the miscut toward $\left[11\bar{2}0\right]$ direction by using the APMOCVD technique. The impact of the off-cut angle on the light emission properties of ZnO films is investigated by using temperature-dependent photoluminescence (TD PL). 

## 2. Materials and Methods

ZnO films were grown on commercially available off-axis 6H-SiC (0001) wafers with off-cut angles of 1.4°, 2°, and 3.5° toward the $\left[11\bar{2}0\right]$ direction and 4H-SiC (0001) with an 8° off-cut angle purchased from SiCrystal (Nürnberg, Germany) and Cree Inc (Durham, NC, USA) respectively. Owing to the limited applicability of 6H-SiC (0001) with an 8° off-cut angle for SiC device technologies, such kind of substrate is, currently, not commercially available. This is a main reason why we used an 8° off-axis 4H-SiC (0001) wafer as a representative of high miscut substrates. At the same time, the equilibrium in-plane lattice constant, *a*, for 4H-SiC is remarkably similar to that of 6H-SiC (both experimentally determined parameters are equal to 3.073 Å) [47]. Such a similarity stipulates a minor effect of the SiC polytype on the ZnO nucleation on the basal plane of hexagonal SiC, since the in-plane lattice mismatch between ZnO and SiC plays a more significant role in the ZnO growth than the corresponding out-of-plane lattice mismatch. Thus, the resulting morphology of the ZnO films on 8° off-axis 4H-SiC (0001) can be, to a large extent, extrapolated to the ZnO growth on 8° off-axis 6H-SiC (0001). To better illustrate the off-cut angle effect on the morphology of ZnO layers, ZnO growth on an on-axis 6H-SiC wafer (from the same suppliers) with a nominally atomically flat surface was also performed.

The choice of SiC wafers as substrates for ZnO growth is above all justified by the fact that these materials have similar structural properties, including lattice constant, thermal expansion coefficient, and space group crystal symmetry [48]. This may result in the formation of ZnO layers with lower defect density and misfit dislocations, as compared to ZnO films grown on sapphire, Si, GaAs, or ScAlMgO_4_ [48]. In addition, the integration of ZnO and SiC technologies is an extremely important step from the practical point of view, since the ZnO/SiC structure has a great potential to be used as isotype or anisotype heterojunction diodes [49]. In this context, it is expected to reach the control of the properties of the ZnO layers by using appropriate vicinal SiC substrates.

ZnO films were deposited by atmospheric pressure metal–organic chemical vapor deposition (APMOCVD) using Zn acetylacetonate (ZnAA, CAS Number 14024-63-6) as a solid-state single source precursor. Growth was performed at the substrate temperature of 500 °C and Ar buffer gas flow rate 50 sccm, which was earlier reported as optimal conditions for the growth of high-quality ZnO films [50]. Prior to growth, the substrates were cleaned by sonication in acetone and ethanol for 10 min and dried by nitrogen flow afterwards. The total growth time was fixed to 30 min. Since the off-cut angle significantly influences the growth rate [28], the film thickness was ranging from 100 nm to 1 μm. 

The microstructure and interface quality of the samples were studied by scanning electron microscopy (SEM) in a Leo 1550 Gemini microscope (Zeiss, Oregon, USA) at an operating voltage ranging from 10 to 20 kV and a standard aperture value of 30 μm. Light emission from the ZnO films was investigated by photoluminescence, which was carried out at the temperature range 4–290 K with a frequency doubled Nd:YVO laser as a continuous wave excitation source, giving a wavelength λ = 266 nm. The luminescence signal was collected and mirrored into a single grating 0.45 m monochromator equipped with a liquid nitrogen cooled Si- Charge-Coupled Device (CCD) with a spectral resolution of about 0.1 meV. The excited area was around 1.5 μm in diameter, providing an excitation density of 2 W/cm^2^. Advanced structural study was performed via reciprocal space mapping (RSM) acquired by high-resolution X-ray diffraction (HRXRD). RSM studies were carried out using a Philips X’Pert high-resolution X-ray diffractometer (Philips X’Pert MPD, Eindhoven, The Netherlands) operating with the Cu Kα1 anode at a fixed voltage and current of 45 kV and 40 mA, respectively. The HRXRD was operated in triple-axis mode, which combines two crystals (Ge 220), four reflection monochromators, and three reflection analyzer crystals.

## 3. Results and Discussion 

### 3.1. Morphology and Crystal Structure of ZnO Films Grown on SiC Substrates with Different Off-Cut Angles

**Figure 1:**
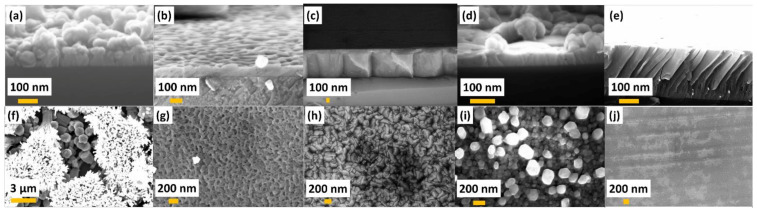
Cross-sectional (**a**–**e**) and top-view (**f**–**g**) SEM images of ZnO layers grown on SiC substrates with different off-cut angles: 0° (**a**,**f**), 1.4° (**b**,**g**), 2° (**c**,**h**), 3.5° (**d**,**i**), and 8° (**e**,**j**), respectively.

Figure 1 presents the cross-sectional and top-view SEM images of as-grown ZnO layers on SiC substrates with off-cut angles ranging from 0° to 8°. The growth onto on-axis SiC substrates resulted in the formation of a polycrystalline film with a columnar structure consisting of misaligned 1 μm-sized rounded (in some cases, hexagonally shaped) columns with overgrown bush-like aggregates (Figure 1a). The top-view SEM image of the same sample is presented in Figure 1f. It is evident that the existing secondary nucleation makes the ZnO film surface quite rough. This top deposition should not be considered as typical and might be regarded as parasitic during growth termination. The columnar structure is a typical morphological feature for mismatched growth systems [51].

Growth on miscut SiC substrates was apparently influenced by the off-cut angle: ZnO films are denser compared to ZnO grown on the on-axis SiC wafer (Figure 1b–e,g–j). The nature of this effect is related to the terrace width dictated by the miscut angle (*θ*). Indeed, a nominally on-axis SiC substrate typically exhibits wide terraces up to 1–5 μm, offering numerous nucleation sites. Consequently, a lot of small grains can grow simultaneously. On the other hand, the terrace width (*TW*) for off-axis SiC substrates is inversely proportional to the tanθ and is decreased with angle increasing [28]:

(1) $$TW=\frac{h}{\tan\theta }$$

where *h* is the step height. Considering that *h* corresponds to two Si-C bilayers (0.504 nm) [28], the terraces’ widths are estimated to be 20.6, 14.4, 8.2, and 3.6 nm for 1.4°, 2°, 3.5°, and 8° substrates, respectively. By adjusting the off-cut angle and increasing the step density, it is possible to limit the undesirable nucleation of ZnO on terraces that causes the formation of grained films. Eventually, this opens the possibility of enhancing the probability of adatom incorporation at step edges and facilitates step-flow growth [28,29]. Therefore, one can anticipate an increase in growth rate with the increasing off-cut angle and improvement of the structural quality of the films. As can be seen from Figure 1h, ZnO film on 2° off-axis SiC demonstrates well-intergrown grains with a mean size of ≈200 nm. The cross-section view of the ZnO film (Figure 1c) showing high thickness uniformity and absence of voids additionally corroborates our initial assumption that the vicinal surface of SiC improves the crystalline quality of ZnO films. It is worth noting that even though the growth process occurs on vicinal surfaces, no obvious inclinations of ZnO grains from the *c*-axis on the substrate with miscut angles up to 3.5° are observed. A possible explanation of this observation is that the real SiC terraces are still too wide to enable all adatoms to reach the step edges, and thus, ZnO concurrently nucleates at steps and on terraces. Indeed, a macrostep bunching can induce the formation of wide atomically flat terraces and high steps on vicinal surfaces of SiC [28,52,53], which is much larger compared to the theoretically predicted values. To some extent, this hinders the step-flow process, leading to incomplete atom incorporation at lattice steps. In contrast, higher off-cut angles better satisfy the step-flow growth conditions and reduce the probability of ZnO nucleation on terraces. As a result, growth on an 8° off-axis 4H-SiC substrate was found to be more unique compared to the other considered cases (Figure 1e,j): columnar growth in this case is less evident, and no individual grains can be identified. The SEM image also suggests that the *c*-axis tilted ZnO film is of much higher structural quality. Although some local imperfections of a film surface can be observed, the resulting ZnO film is exceptionally smooth. To sum up, one can argue that only the SiC substrates with a high off-cut angle (8°) substantially affect the film morphology. Meanwhile, the growth of ZnO on SiC substrates with smaller off-cut angles is mainly determined by the nucleation processes on terraces, and thus, no strong off-cut angle effect is expected. Indeed, in the case of wide terraces, the early stages of the film growth are governed by several key processes: (i) random deposition on the SiC surface, (ii) random terrace diffusion of just-arrived species, and (iii) and the formation of stable nuclei. As a result, the random character of the selected processes during film growth on SiC with small off-cut angles is more important than the off-cut angle changes in determining the overall morphology of ZnO films. However, since the terrace width is changed with increasing the off-axis angle, we expect case-to-case variations of defect density. In this regard, temperature-dependence photoluminescent spectroscopy (discussed later) is an extremely sensitive technique to monitor defect-related optical transitions and therefore is highly informative to explore the quality of ZnO films.

**Figure 2:**
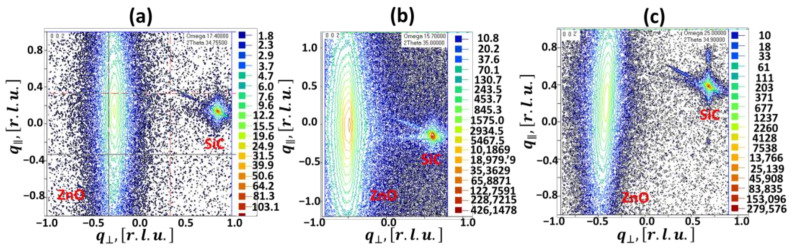
X-ray reciprocal space maps (RSMs) around the (002) reflection measured for ZnO films grown on SiC substrates with different off-cut angles: 0° (**a**), 2° (**b**), and 8° (**c**), respectively. Note: A 2° off-axis SiC substrate is chosen as a representative of SiC with low off-axis orientation, while 8^°^ off-axis SiC is regarded as a highly misoriented substrate. [r.l.u.] is reciprocal lattice units. Small spots correspond to SiC substrates. $q_{⊥}$ is the path in the *q*-space for the 2*θ*-*ω* scan, whereas $q_{∥}$ is the path in the *q*-space for rocking curve scan.

To shed more light on the effect of the off-cut angle on the structural quality of ZnO films grown on vicinal surfaces of SiC, we also performed high-resolution X-ray diffraction studies. The results showed that all grown films have a wurtzite hexagonal structure with (002) preferential orientation. Figure 2 illustrates the reciprocal space maps (RSMs) of the symmetric (002) reflection for ZnO films grown on nominally flat and off-axis SiC substrates. From Figure 2, it is clearly seen that the mosaic spread ($\Delta q_{∥}$) for ZnO on 8° off-axis SiC (Figure 2c) is much smaller compared to that for ZnO on on-axis SiC and 2° off-axis SiC (Figure 2a,b, respectively). This indicates the better crystalline quality of ZnO on 8° off-axis SiC, which additionally supports the results of morphology analysis. Furthermore, we noticed that the elongated ellipsoidal spot on RSM for ZnO on 2° off-axis SiC (Figure 2b), which represents the case of low-angle off-axis substrate orientation, is asymmetrical. Indeed, a more detailed look at the 2*θ*-*ω* spectrum reveals that the (002) peak is a result of two overlapping peaks: one at 34.4845° and another at 34.5228°. Such a peak splitting may originate from the co-formation of two types of domains due to the growth on SiC terraces, from one side, and a step-edge nucleation process, from another side. This speaks in favor of the mixed growth mode for ZnO on a low-angle off-axis substrate.

### 3.2. Photoluminescence of ZnO Films Grown on SiC Substrates with Different Off-Cut Angles

**Figure 3:**
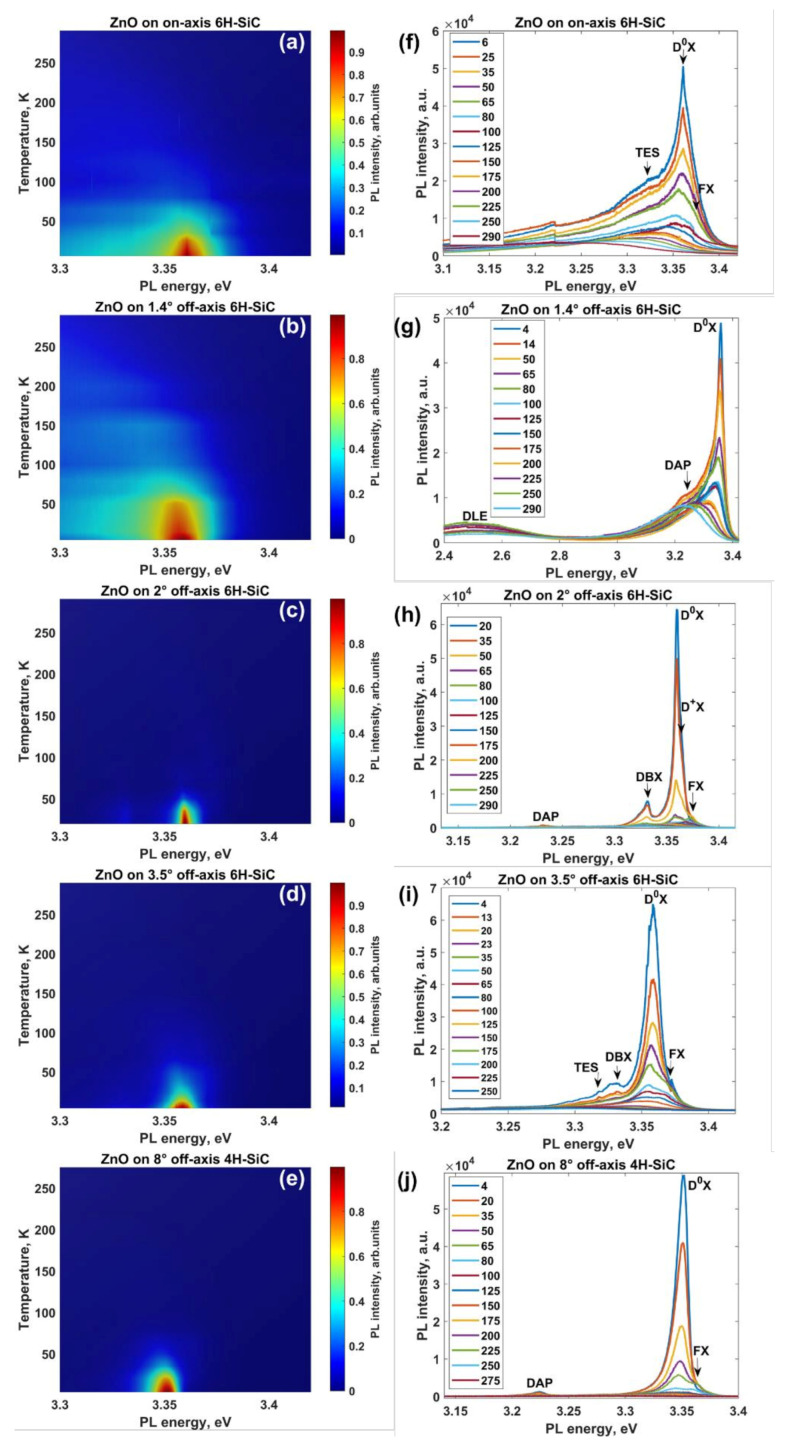
PL contour plots of temperature-dependent ultraviolet emission (**a**–**e**) and corresponding PL spectra (**f**–**j**) of ZnO films grown on SiC substrates with different off-cut angles: 0° (**a**,**f**), 1.4° (**b**,**g**), 2° (**c**,**h**), 3.5° (**d**,**i**), and 8° (**e**,**j**), respectively. Since the low-intensity spectral features with peak energies smaller than 3.3 eV cannot be easily seen against the blue background of PL contour plots, we did not visualize the spectral range from 2.4 to 3.3 eV, as that is less informative for readers. Note: Spectral features observed for ZnO on on-axis 6H-SiC in the range 3.2 to 3.25 eV are not luminescence features but originate most likely from a change within the grating system of the dispersing element.

Off-cut angle-induced changes in the material quality of ZnO are reflected in the optical properties of ZnO layers (Figure 3).

A quick look at the contour plots of the temperature-dependent photoluminescence spectra of all considered samples confirms that the off-cut angle significantly affects the appearance of the excitonic emission and luminescence thermal quenching (Figure 3a–e). For all films, when the temperature is increased systematically, the intensity of the dominant emission line decreases due to the thermal dissociation of bound excitons (Figure 3f–j). At moderate and high temperatures, the largest fraction of bound excitons is thermalized, and a small shoulder from the high-energy side of the bound exciton peak becomes more distinct. This spectral feature is related to free exciton emission. However, no clear free exciton emission band was resolved in the spectra of the ZnO films grown on a 1.4° off-axis 6H-SiC substrate (Figure 3g), which can be explained by the inhomogeneous broadening of the PL spectra [54] induced by the ionized impurity scattering, wide variations in donor binding energies, and/or even strain [55]. 

To get deeper insights into the optical transitions in ZnO films grown on SiC substrates with off-cut angle ranging from 0° to 8°, we will look at this issue in more depth in the following sections of the paper.

#### 3.2.1. Off-Cut Angle Effect on the Low-Temperature PL

**Figure 4:**
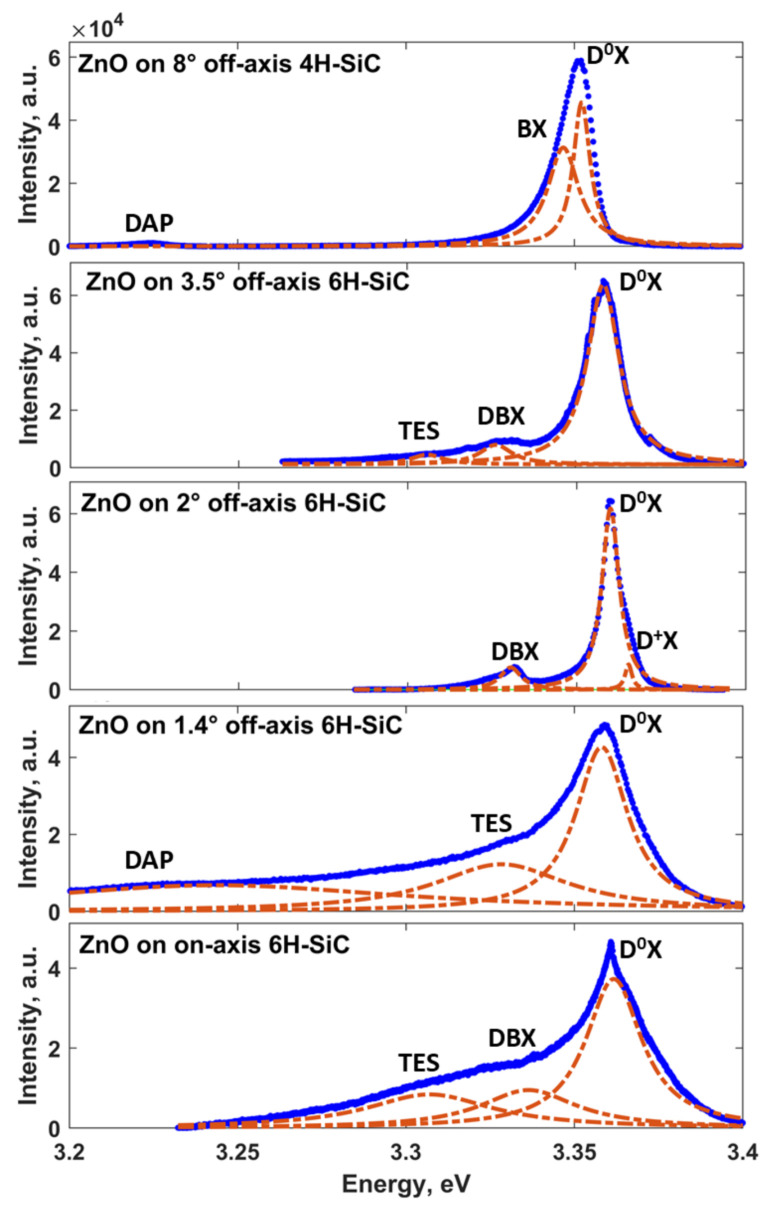
Low-temperature PL spectra (at 4 K) with peak assignment for ZnO films grown on SiC substrates with different off-cut angles: the off-axis angle increases from the bottom panel to the top panel. Blue solid lines are experimental data, while the dash-dot orange curves are the fitting Lorentzian curves. At this temperature, the large fraction of electrons is localized at donor centers related to dopants or defects, and thus, PL spectra are dominated by emissions from bound excitons. Therefore, no FX emission is observed in the low-temperature regime. Fitting errors are estimated to be 2.648%, 2.846%, 1.591%, 1.29%, and 1.632% for ZnO films grown on SiC substrates with different off-cut angles: 0°, 1.4°, 2°, 3.5°, and 8°, respectively.

The photoluminescence spectra for all samples were first measured at low temperature (4 K). As shown in Figure 4, in all cases, the PL signal is dominated by donor-to-bound excitons (D^0^X) emission. The most intense peaks are located at 3.3617, 3.358, 3.3601, 3.3582, and 3.3517 eV for ZnO on 0°, 1.4°, 2°, 3.5°, and 8° off-axis substrates, respectively. According to Meyer’s classification, these excitonic lines can be assigned to neutral impurity bound excitons I_5_, I_8/8a_, I_7_, I_8/8a_, and I_10_, respectively [56]. In the case of the ZnO reference sample on a nominally on-axis SiC substrate, the I_5_ peak is asymmetrically broadened and has a low-energy shoulder, including two additional spectral features at 3.3116 and 3.3374 eV. These two can be attributed to a two-electron satellites (TES) transition of the I_5_ exciton [56,57] and emission related to structural defect bound excitons (DBX) [56,57], respectively. The analysis of the low-temperature PL spectrum of the ZnO film grown on the 1.4° off-axis substrate showed that this sample is most defective as evidenced by the observation of the deep level emission (DLE) peak located at 2.4961 eV that is absent for other samples (Figure 4g). The observed visible emission may be due to the electron transition from the conduction band to oxygen antisite (O_Zn_) defect levels [58,59]. In addition, the dominant I_8/8a_ peak has a small tail extending to long wavelength (low-energy side) with two resolved components at 3.2438 and 3.3277 eV. The first peak can be attributed to donor–acceptor pair (DAP) emission [60], while the second feature corresponds to the TES transition of the I_8/8a_ exciton [56,57]. As was mentioned in the previous section, structural properties of the ZnO films are being improved with the miscut angle increasing. As a direct consequence of that, the PL spectra of ZnO films grown on substrates with larger off-cut angles have a more pronounced fine structure without high-intensity energy tails that mask the electronic transitions. We believe that this is indicative of the better optical quality of ZnO films caused by the beneficial effect of the growth on vicinal surfaces. In this regard, additionally to the already mentioned I_7_ line, two separate emission peaks are observed at ≈3.2320 and 3.3301 eV for ZnO on 2°-off-axis SiC, which likely correspond to DAP-related transition and excitons bound to structural defects. Moreover, from the high-energy side of the most intense D^0^X emission peak, a new spectral feature at 3.3649 eV can be also distinguished. Since the corresponding energy is smaller than the typical free-exciton energy region, we can ascribe this peak to ionized donor bound excitons (D^+^X) [57] rather than to free-exciton (FX) emission. 

Moving to the ZnO film grown on the 3.5° off-axis SiC, we noticed the presence of TES transition and DBX emission related to structural defects. It is worth noting that the analysis of the PL spectra of the ZnO samples on substrates with miscut angles ranging from 0° to 3.5° revealed the existence of optical transitions involving structural and/or antisite defects with deep defect energy levels. This suggests that the corresponding growth conditions (namely, substrate morphology) promote the defects generation during the film formation. Another picture takes place in the case of the ZnO film grown on 8° off-axis 4H-SiC. More specifically, we did not observe any features related to structural defects. The PL spectrum comprises only three distinctive components related to DAP emission (at 3.2228 eV), bound exciton (at 3.3442 eV) of unknown identity, and deep neutral donor bound exciton I_10_ at 3.3517 eV. 

One more important argument that speaks in favor of our assumption that the substrate miscut angle improves the optical quality of the ZnO samples can be extracted from the analysis of the Full Width at Half Maximum (FWHM) of the D^0^X emission. Indeed, FWHM values of the most intense excitonic peaks for ZnO on 0°, 1.4°, 2°, 3.5°, and 8° off-axis substrates are found to be 19, 19, 12, 11, and 9 meV. The observed decrease of FWHM with miscut angle increase implies smaller energy landscape fluctuations in ZnO due to reduced structural defects’ density and a more uniform distribution of exciton binding energies.

#### 3.2.2. Off-Cut Angle Effect on the Room-Temperature PL

**Figure 5:**
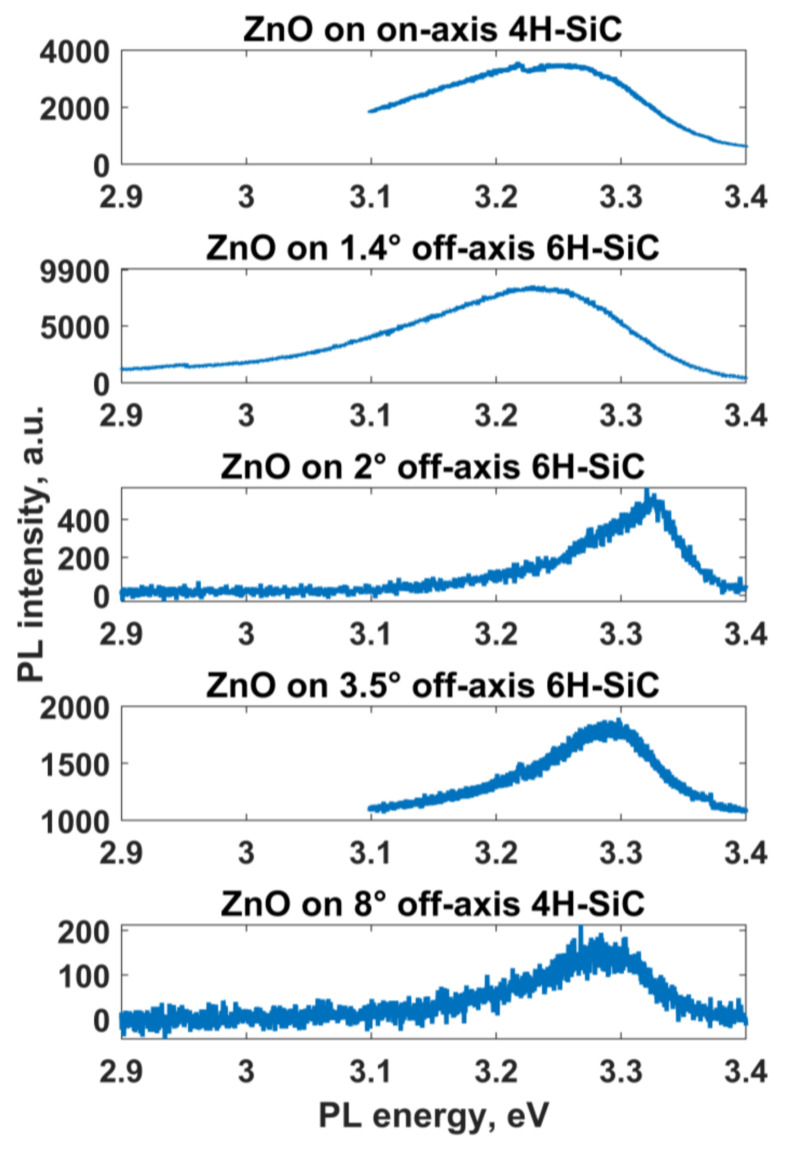
Room temperature PL spectra collected from ZnO films grown on SiC substrates with different off-cut angles: off-axis angle increases from the top panel to the bottom panel.

Figure 5 displays the room-temperature PL spectra of the ZnO layers grown on 0°, 1.4°, 2°, 3.5°, and 8° off-axis substrates. It was revealed that the near-band edge (NBE) emission from the ZnO layers grown on 0° and 1.4° SiC is significantly redshifted compared to that of ZnO on 2°, 3.5°, and 8° SiC, and this phenomenon can be interpreted as a result of strong exciton–phonon interactions associated with surface defects [61]. This further corroborates the analysis contained in the previous section.

#### 3.2.3. Temperature Dependences of PL Parameters

**Figure 6:**
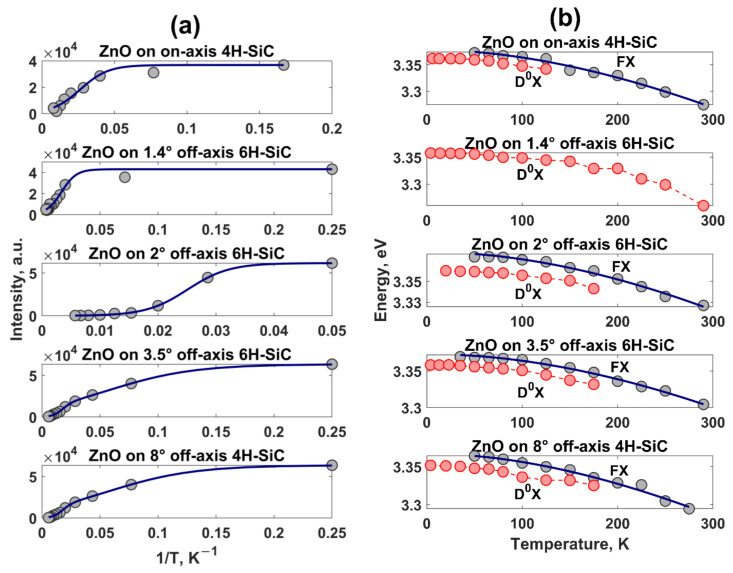
(**a**) The intensity of donor-to-bound excitons (D^0^X) emission peak as a function of inverse temperature (filled gray circles) and corresponding fitting solid blue curves (Equations (2) and (3)) for ZnO films grown on SiC substrates with different off-cut angles. The goodness of Arrhenius fit (R^2^) is predicted to be 0.9519, 0.9648, 0.9998, 0.9996, and 0.9999 for ZnO films on 0°-6H-SiC, 1.4°-6H-SiC, 2°-6H-SiC, 3.5°-6H-SiC, and 8°-4H-SiC, respectively. (**b**) The energy position of the FX peak (filled gray circles) as a function of temperature and the corresponding fitting solid blue curves (Equation (4)). The goodness of Varshni fit (R^2^) is predicted to be 0.9891, 0.9898, 0.9937, and 0.9842 for ZnO films on 0°-6H-SiC, 2°-6H-SiC, 3.5°-6H-SiC, and 8°-4H-SiC, respectively. We also depicted the temperature dependence of the D^0^X emission peak. The off-axis angle increases from the top panel to the bottom panel.

Figure 6a demonstrates the dependences of the intensity of the D^0^X peak with the reciprocal of temperature for all considered films. Generally, the thermal quenching of the D^0^X emission can be explained by the following one-term or two-term Arrhenius relationships [62,63,64]:

{(2)IT =I01+Ae−EakBT(3)IT =I01+A1e−Ea1kBT+A2e−Ea2kBT

where $E_{a}$, $E_{a1}$, and $E_{a2}$ are the activation energies, $k_{B}$ is the Boltzmann constant, $I_{0}$ is the D^0^X peak intensity at 0 K, *T* is the temperature, and A, $A_{1}$, and $A_{2}$ are constants. It was found that the temperature dependence of D^0^X intensity for ZnO samples grown on substrates with 0°, 1.4°, and 2° off-cut angles can be well fitted by the one-term Arrhenius Equation (2).

The best fitting gives the following activation energies: 8.97 ± 0.58 meV, 13.37 ± 0.66 meV, and 24.11 ± 0.07 meV, respectively. It is obvious that the increase of the miscut angle gives rise to an increase in activation energy. This means that the dissociation process of D_0_X is slower for ZnO samples grown on vicinal surfaces compared to ZnO film on a nominally on-axis SiC wafer. Such a finding can be interpreted as a result of the smaller number of structural defects, which may strongly affect the bound excitons [61]. It is interesting to note that the temperature evolution of D_0_X intensity for ZnO films grown on SiC substrates with higher off-cut angles (3.5° and 8°) can be described by a biexponential Arrhenius model (Equation (3)), which implies the presence of two non-radiative recombination channels for D_0_X emission. Our results show that the best-fitting $E_{a1}$ and $E_{a2}$ parameters are 2.32 ± 0.02 (at low temperature) and 21.67 ± 0.31 meV (at high temperature) for ZnO on 3.5° 6H-SiC and 5.93 ± 0.02 (at low temperature) and 47.30 ± 0.47 meV (at high temperature) for ZnO on 8° 6H-SiC, respectively. Further analysis of the temperature-dependent PL spectra of as-grown ZnO films revealed also that D_0_X and FX emission peaks experience a gradual red-shift with increasing temperature (Figure 6b). The observed FX energy shift can be well described by the Varshni Equation [65]:

(4) $$E\left(T\right)\;=E\left(0\right)-\frac{\alpha T^{2}}{\left(T+\beta \right)}$$

where $E\left(0\right)$ is the transition energy at 0 K, *α* and *β* are the constants, which are associated with the exciton–phonon interaction and to the Debye temperature, respectively [66]. The values of the parameter α for ZnO films on SiC with 0°, 2°, 3.5°, and 8° off-axis angles were 1.59 ± 0.03, 1.10 ± 0.02, 1.47 ± 0.02, and 1.10 ± 0.02 meV·K^−1^, while *β* values were 1020 ± 26, 1487 ± 40, 1518 ± 36, and 920 ± 28 K, respectively. The estimated best-fit parameters for ZnO films are in line with the previous reported values for ZnO film and nanostructures [61,67]. It strikes the attention that *α* and *β* parameters for ZnO on 8°-6H-SiC are close to the literature data for ZnO crystals [67,68], indicating a high structural quality of formed ZnO film.

## 4. Conclusions

We have deposited ZnO films onto flat and vicinal SiC (0001) substrates using the APMOCVD technique. By changing the off-axis angle from 0° to 8°, we showed a principal possibility to change the structural quality of ZnO layers: from a columnar structured film to a high-quality *c*-axis tilted ZnO layer without a grained structure. An off-axis angle of 8° was identified to promote the step-flow growth of ZnO layers with improved optical properties and no evidence of structural defects-involved electronic transitions. A strong correlation between film morphology and optical properties was revealed. The analysis of temperature-dependent PL spectra showed a dominated peak of D^0^X emission at low temperatures followed by prevailing FX emission at high temperatures. No FX emission for ZnO films grown on 1.4° off-axis 4H-SiC was observed, which is attributed to inhomogeneous broadening of the PL spectra. Two nonradiative recombination channels for the D^0^X emission were identified for ZnO films on SiC with large off-cut angles. The Varshni model was applied to describe the temperature dependence of FX energy. Derived parameters corresponding to exciton–phonon interaction and Debye temperature are consistent with those of ZnO single crystal, suggesting the beneficial effect of 8° off-axis angle on the optical properties of ZnO layers. The present work provides a holistic understanding of the nature of the radiative recombination in ZnO grown on off-axis SiC substates, thereby boosting the development of new-type isotype and anisotype ZnO/off-axis-SiC heterojunctions, which are promising for various optoelectronic applications, including UV light emitting diodes, solar cells, and photodetectors.

## Data Availability

The data that support the findings of this study are available within the article.
